# Holidays

**Published:** 1897-09-11

**Authors:** 


### Holidays.

There are few respects in which the Victorian age has witnessed greater changes than with regard to holidays. In the medical profession, when the Queen came to the throne, no country general practitioner ever thought of being absent from his work. It would have been an offence against public opinion if he had failed to be on the spot, not because he was wanted, but because he might be. Mrs. Jones, if she had heard that her customary medical attendant had gone to London for a week, would have felt herself bound to fall ill incontinently, and to send for some other practitioner, merely as a lesson to the delinquent. The useful gentlemen who are now described by country housemaids as "local demons" had not yet come into existence; for, in the lack of demand, there would of course be no supply. Prior to railways, of course, both time and cost were of the essence of the question, and the mere physical difficulty of leaving home had a tendency to confirm the limpet-like characters of the men of the day. They jogged on in a routine of duty, supplementing the medical attainments of their youth with such "experience" as their daily work had brought, remote altogether from professional progress and from the course of scientific research, but yet highly valued and valuable members of the societies in which they dwelt. The retrospect affords a strange contrast to the conditions with which we are now familiar. The old doctor was apt to be more or less a fossil; the new one may perhaps sometimes go astray in the direction of a too ardent pursuit of novelty.

The change in the habits of doctors, however, is merely typical of that which has come over the lives of our people generally; and it is perhaps an open question to what extent we have been gainers. Occupation, from the highest intellectual work to the most trivial mechanical routine, is now spoken of as if it were something certain to be injurious if too long continued or too zealously pursued; and everybody, from the maid of all work in a lodging-house to the ruler of the destinies of an empire, is supposed to stand in need of holidays and of recreation, and to be unable to continue the accustomed calling, whatever it may be, without frequent periods of diversion of mind to some other. The waiter, who spent his chance holiday in assisting a brother of his craft, belonged to a type which has ceased to exist; and our waiters nowadays are to be seen taking their ease at Margate, or even at Brighton. We hear of the need for mental rest which is experienced by persons who never did a piece of decent brainwork in their lives, and of the need for change which is felt by others whose dulness would seem to furnish them with armour of proof against any influence from their surroundings. It follows from the universality of holiday-making that much of it is made in a hurry, and it may fairly be doubted whether, in any sense, the profit is then commensurate with the exertion and the cost, which latter often severely taxes the resources of those by whom it is incurred. The persons who are induced to proceed on scrambling tours for a week or for a fortnight into countries of which they know neither the history nor the language, who spend their days partly in dusty railway carriages and partly in being dragged in procession from one sight which they do not understand to another, should fairly ask themselves, as soon as the expedition is over, whether they have gained anything of real value. If they have not, the experience should teach them to eschew similar extravagances for the future, and to take their holidays in circumstances more familiar to them, and more advantageous, because more restful to their minds. For most young people of small means, for clerks and shop assistants and so forth, we apprehend that the best possible form of holiday is one which renews the sympathies and the attachments of home, and that a sojourn at the parental cottage in Devonshire or in Norfolk is far more likely to be useful, both physically and morally, than a few days in (say) "lovely Lucerne." The opportunities for holiday making in England itself have been immensely enlarged, for both sexes, by the popularity of the bicycle, but even this requires a word of warning. The object of a holiday is recuperation, and recuperation cannot occur when waste is permitted to exceed repair. Muscles are capital, and their daily output of energy is expenditure, which should never be suffered to exceed, and which should not as a rule even too nearly approach, the income which they are calculated to afford. The bicyclist on a tour should be tired enough to sleep soundly, and not tired enough to be weary or distressed upon the morrow.

